# Additive Manufacturing of Shape-Changing Printlets via Powder-Based Extrusion 3D Printing of Natural Cellulose and Polyvinyl Alcohol

**DOI:** 10.3390/polym18030380

**Published:** 2026-01-30

**Authors:** Kasidit Dokhom, Pensak Jantrawut, Pattaraporn Panraksa, Suruk Udomsom, Wirongrong Tongdeesoontorn, Baramee Chanabodeechalermrung, Pornchai Rachtanapun, Tanpong Chaiwarit

**Affiliations:** 1 Department of Pharmaceutical Sciences, Faculty of Pharmacy, Chiang Mai University, Chiang Mai 50200, Thailand; kasidit_dokh@cmu.ac.th (K.D.); pensak.j@cmu.ac.th (P.J.); pattaraporn.pan@cmu.ac.th (P.P.); baramee.c@cmu.ac.th (B.C.); 2 Center of Excellence in Agro Bio-Circular-Green Industry (Agro BCG), Agro-Industry, Chiang Mai University, Chiang Mai 50100, Thailand; pornchai.r@cmu.ac.th; 3 Department of Electrical Engineering, Faculty of Engineering, Chiang Mai University, Chiang Mai 50200, Thailand; suruk.u@cmu.ac.th; 4 Office of Research Administration, Chiang Mai University, Chiang Mai 50200, Thailand; 5 School of Agro-Industry, Mae Fah Luang University, 333 Moo 1 Tasud, Chiang Rai 57100, Thailand; wirongrong.ton@mfu.ac.th; 6 Research Center of Innovative Food Packaging and Biomaterials Unit, Mae Fah Luang University, 333 Moo 1 Tasud, Chiang Rai 57100, Thailand; 7 Faculty of Agro-Industry, Chiang Mai University, Mae-Hea, Mueang, Chiang Mai 50100, Thailand

**Keywords:** powder melt extrusion, smart materials, short fiber, cellulose, shape changing, levodopa

## Abstract

Powder melt extrusion (PME) represents an alternative approach for personalized oral dosage forms. Furthermore, the utilization of agricultural waste has gained increasing attention because it helps reduce pollution from waste. This study investigated cellulose powders and short fibers from agricultural waste as supporting materials for the PME-based production of shape-changing levodopa printlets. Formulations containing cellulose powder (CP), cassava short fiber (CSF), and pineapple short fiber (PSF) demonstrated successful printing. The selected formulations were characterized for morphology, thermal transitions, crystallinity, shape-changing behavior, and drug release. CSF demonstrated superior printability, enhanced shape recovery, and the greatest reduction in crystallinity, supporting amorphous solid dispersion formation. Levodopa-loaded printlets showed uniform and high drug content. The formulation containing 5% CSF and levodopa exhibited the fastest initial release, attributed to its low crystallinity and Super Case II transport mechanism. Overall, this study highlights the feasibility of using natural cellulose as an additive in PME to develop sustainable, shape-changing drug delivery systems and advances PME knowledge by integrating agricultural waste derived cellulose fibers with levodopa processing that provide new insight into the material–process–performance relationship in PME systems.

## 1. Introduction

The recent development of drug formulations and delivery systems progressively focuses on utilizing eco-friendly materials and personalized dosage forms [1]. The agricultural sector in Thailand represents a major part of the country’s economy, at around 46% of total production areas. However, it also generates a large amount of agricultural waste, such as durian rind, pineapple leaves, and cassava peels [2,3]. Typically, these wastes are not utilized and are often burned to clear land for the next cultivation cycle. The smoke and particulate emissions from burning contribute to air pollution, such as particulate matter with a diameter of less than 2.5 micron (PM2.5) [3]. Moreover, some agricultural waste that is not burnt becomes biowaste [4]. These agricultural wastes can add value as biopolymers or reinforcement fillers for supporting both environmental sustainability and reduced dependence on synthetic polymers [5,6] in alignment with the United Nations Sustainable Development Goals (SDGs).

Cellulose is widely applied as a raw material in various industries, including food, beverage, and paper production [7,8]. In the pharmaceutical industry, cellulose and its derivatives have been applied in drug delivery systems. For example, hydroxypropyl methylcellulose (HPMC) and hydroxypropyl cellulose (HPC) have been utilized in the manufacturing of oral films and controlled-release tablets [9]. Moreover, the cellulose extracted from agricultural residues provides good mechanical properties and polymer compatibility for stimuli-responsive or shape-changing materials [10]. In addition, fiber bundles processed into short fibers [11] have also gained attention to support the strength of formulations in 3D printing applications [11,12]. In previous studies, inks combined with cellulose have been used to enhance the structural flexibility of hydrogels in 3D printing [13].

Additive manufacturing (AM), especially three-dimensional (3D) printing, has become a significant technology, as it enables the production of personalized dosage forms with adjustable dose, geometry, internal structure, and release profiles tailored to individual patients [14]. This approach supports improved treatment effectiveness, patient adherence, and overall quality of life. Moreover, the concept of four-dimensional (4D) printing, which involves 3D structures capable of changing shape in response to external stimuli, such as temperature and humidity, is gradually gaining attention in drug delivery due to controlled drug release through shape transformation [15,16]. Nevertheless, the existing studies indicate that the application of powder melt extrusion (PME) in pharmaceutical 4D printing concepts is a relatively new area, and few studies have been conducted.

Three-dimensional printing technologies have advanced rapidly. Among these, PME, which is a modified form of hot-melt extrusion (HME), utilizes powdered materials instead of filament. The powdered material is fed into a print head with a rotating single-screw, heated, and extruded through the nozzle to print layer by layer. The advantages of PME include the use of powder raw materials without the need for pellet or filament preparation, reducing processing steps [17]. Moreover, PME can enhance the solubility of poorly water-soluble drugs by forming amorphous solid dispersions (ASDs) through the melting and mixing of polymer and drug powders. PME reduces crystallinity, leading to improved dissolution and bioavailability [18]. These advantages make PME an interesting technique for the development of drug formulations.

Polyvinyl alcohol (PVA) is a widely used polymer in the pharmaceutical field and 3D printing due to its biocompatibility, water absorption, and swelling capacity [19]. These properties make it suitable for shape-morphing behavior when exposed to aqueous environments at certain pH, such as the gastrointestinal tract [20]. Despite the limited use of PVA in PME, PVA has been employed in HME to provide appropriate melt viscosity for 3D printing because PVA is water-soluble [19], objects printed solely from PVA can maintain their structural integrity in aqueous media, such as simulated gastric fluid, only for a short period. In contrast, combining PVA with cellulose derived from agricultural waste can improve mechanical strength [21] and enhance the ability of the printed objects to retain their structure for a longer duration in simulated gastric fluid. This property could be exploited to modulate drug release from the printed objects.

Levodopa is the most effective medicine used in the treatment of Parkinson’s disease. However, patients often experience motor fluctuations, including predictable wearing-off periods, unpredictable off episodes, delayed-on, and no-on phenomena. Motor fluctuations emerge from multiple factors, such as the short half-life of levodopa and the poor aqueous solubility of levodopa, which affects gastrointestinal absorption [22]. Three-dimensional printing drug delivery systems help address these limitations by developing controlled-release formulations and enhancing the solubility of levodopa through ASDs [18]. Designing dosage forms with size- or shape-changing capabilities can prolong gastric residence time, thereby enabling gradual absorption of levodopa and contributing to prolonged release for controlled or sustained drug delivery [23]. In particular, levodopa is a drug with a narrow therapeutic index [24]. Therefore, controlled-release formulations are important for maintaining stable drug concentrations and reducing fluctuations in drug levels. Patients have a lower risk of adverse effects associated with excessive or of treatment failure with insufficient dosing. Moreover, levodopa exhibits sufficient thermal stability in PME [25]. Therefore, levodopa is suitable for use as a model drug to allow the process to be adapted for future applications, demonstrating the capability of the printing process to precisely produce the desired dosage.

Previous studies have incorporated cassava short fibers into powder melt extrusion. This study aims to develop and investigate the 3D printing of cellulose materials derived from agricultural waste with PVA via the powder melt extrusion process to fabricate shape-changing 3D structures containing levodopa. The effects of cellulose type and composition on printing behavior, shape-morphing capability, and drug release profiles of levodopa are examined. The present study expands the scope of previous research by investigating various short fibers derived from agricultural waste, in addition to a comparative evaluation with cellulose powder. The subject of this study is defined by focusing on the previously unexplored role of short fibers from various agricultural waste sources in governing printability, solid-state transformation, and shape-changing behavior of levodopa containing produced by PME. This study expects that these short fibers could enhance material performance and influence the drug release behavior of printlets produced by powder melt extrusion.

## 2. Materials and Methods

### 2.1. Procedure

**Figure 1:**
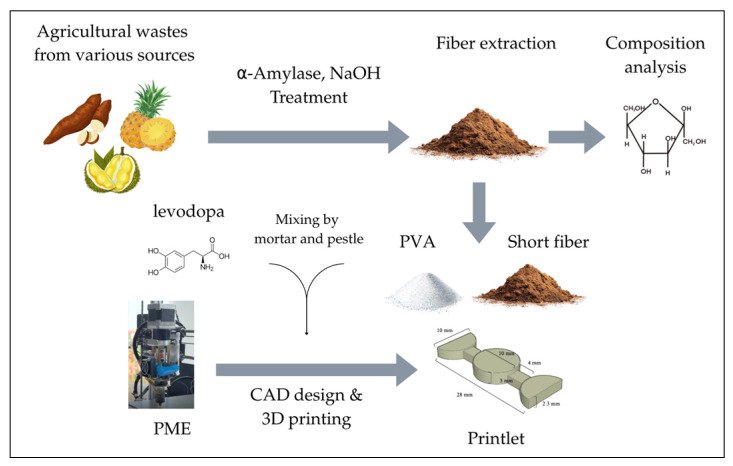
Schematic illustration of the main work stages in the preparation of short cellulose fibers to obtain the printlet.

The procedure of this study is shown in Figure 1. Agricultural waste was first processed into short cellulose fibers, which were blended with PVA to form a bio-hybrid (polymer and fibers). Levodopa was incorporated, followed by fabrication of shape-changing printlets using PME. The obtained printlets were subsequently characterized.

### 2.2. Materials

Polyvinyl alcohol (PVA) Mowiol^®^ 4-88, partially hydrolyzed PVA, and MW ~31,000 g/mol was procured from Sigma-Aldrich^®^ (St. Louis, MO, USA). Cellulose powder (CP) was procured from HiMedia Laboratories Pvt. Ltd. (Nashik, India). Cassava pulp, hemp stem, durian peels, and pineapple leaves were obtained from the Faculty of Agro-Industry, Chiang Mai University (Chiang Mai, Thailand). Ethanol was acquired from RCI Labscan, Ltd. (Bangkok, Thailand).

### 2.3. Working Methods

#### 2.3.1. Obtaining Short Fibers

Pineapple leaves, cassava pulp, hemp, and durian peels were thoroughly washed to remove contaminants, then cut into approximately 1-inch lengths. The cleaned materials were dried in a hot air oven (UN30, Memmert GmbH + Co. KG, Schwabach, Germany) at 70 °C for 24 h to remove the residual starch by adding 100 g of raw material with 1000 mL of distilled water and adjusting the pH to 6.5–7.0 using a 3% sodium hydroxide (NaOH) solution. Then, the mixture was heated to 90 °C for 20 min before adding α-amylase (20 IU/mL) to remove the residual starch. Consequently, the mixture was filtered using a vacuum filtration system before drying at 70 °C for 5 h. Finally, the obtained starch-free fibers were treated with chemicals to eradicate remaining hemicellulose and lignin utilizing 12% NaOH solution at a 1:20 ratio, followed by heating at 85 ± 5 °C for 3 h, with stirring at 500 rpm and drying at 70 °C for 5 h. The obtained fibers were ground using a hammer mill and sieved using a sieve shaker (Sieve, Retsch Laboratory Equipment, Haan, Germany) through sieve No.325 to obtain a 45-µm particle size. The obtained short fibers were cassava short fiber (CSF), pineapple short fiber (PSF), durian short fiber (DSF), and hemp short fiber (HSF). The extraction yield was calculated using Equation (1). The selected short fibers were analyzed for their chemical composition, as follows:

(1) $$\mathrm{Extraction}\;\mathrm{yield}\;(\%)\;=\;\frac{W_{2}}{W_{1}}\times 100$$

where

W_1_ is the weight of material before extraction (g).
W_2_ is the weight of short fiber after extraction (g).

#### 2.3.2. Obtaining Bio-Hybrids (Polymer and Fibers)

PVA was crushed to a fine powder by a blender machine (PG-ECO, SGE Co., Ltd., Bangkok, Thailand) and sieved through sieve No.325. PVA fine powder was blended with cellulose powder or short fibers through the geometric dilution method with a mortar and pestle in a defined ratio, as shown in Table 1.

#### 2.3.3. 3D Printing

**Figure 2:**
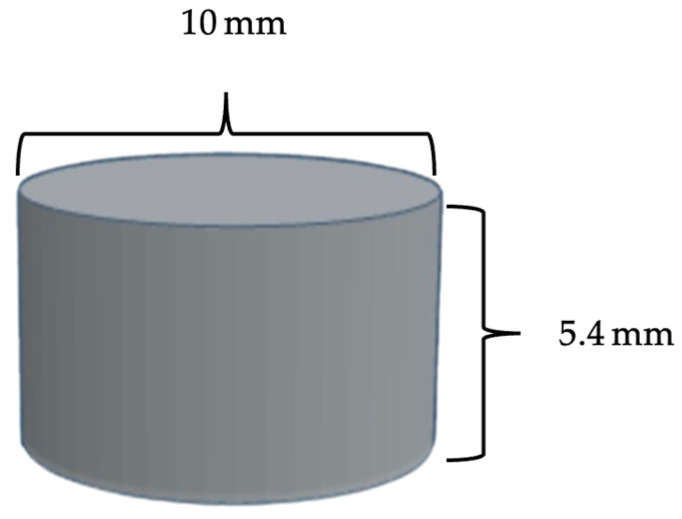
A prototype in the tablet-like shape.

A prototype was designed in a tablet-like shape to evaluate printability. The prototype has a diameter of 10.00 mm and a height of 5.40 mm (Figure 2). The prototype was created using AUTODESK^®^ FUSION 360™ (version 2.0.16985; Autodesk Inc., San Rafael, CA, USA). The computer aid design (CAD) file was exported to standard triangulation language (STL) format and processed with a slicing program. Subsequently, the prototype was printed using a PME printer developed by the Biomedical Engineering Institute of Chiang Mai University, as formulation Table 1 [12]. Each prototype was printed layer-by-layer through a 0.80 mm nozzle at 195 °C with an extrude rate of 5.00 rpm and printing speed of 5.00 mm/s onto a polyetherimide (PEI) build plate at 65 ± 5 °C to prevent premature solidification of the polymer.

**Figure 3:**
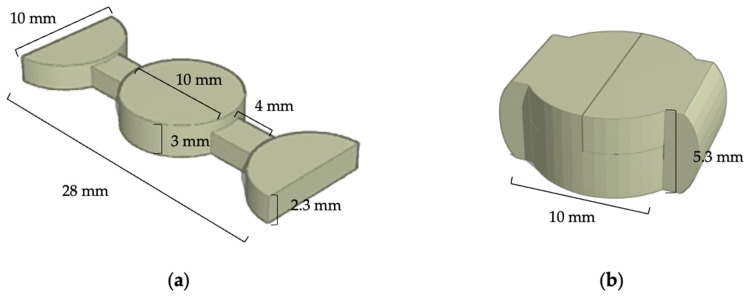
Design for the printlet using AUTODESK^®^ FUSION 360^TM^ Software (**a**), and the fixed shape after using heat (**b**).

The selected formulations were added with levodopa in a concentration of 10% w/w, as shown in Table 2. All formulations were printed as a designed printlet consisting of a central cylinder (Ø 10.0 mm × 3.0 mm height), a 4.0 mm joint, and two half-cylinder lobes (Ø 10.0 mm × 2.3 mm height), giving an overall length of 28.0 mm and width of 10.0 mm, which was custom-designed using AUTODESK^®^ FUSION 360™ (Figure 3). The printlet was printed on a PME system under the same conditions as the prototype.

### 2.4. Characterization

#### 2.4.1. Identification of Cellulose Components

(a) Holocellulose content

Holocellulose, representing the total polysaccharide fraction, was determined following the Method of Wood Chemistry (TAPPI Section, 10 January 1946). An extractive-free sample (2.5 g) was transferred to a 250 mL Erlenmeyer flask containing 150 mL of distilled water, 0.2 mL of glacial acetic acid, and 2 g of sodium chlorite (NaClO_2_). The mixture was maintained at 70 °C in a water bath, with additional portions of 0.2 mL of acetic acid and 1 g of NaClO_2_ added at hourly intervals over a 4 h period. After completion, the reaction mixture was cooled to below 10 °C for 30 min and subsequently filtered through a filtering crucible. The residue was thoroughly washed with ice-cold distilled water, followed by acetone (≈100 mL). The obtained holocellulose was dried at 105 °C for 24 h, and its content was calculated using Equation (2).

(2) $$\mathrm{Holocellulose}\;(\%)=\frac{\mathrm{Holocellulose}\;(g)}{\mathrm{Extractive}\text{-}\mathrm{free}\;\mathrm{wood}\;(g)+\mathrm{Extracrtive}\;(g)}\times 100$$

where the extractive was calculated using Equation (3).

(3) $$\mathrm{Extractive}\;(g)=\frac{\;\mathrm{weight}\;\mathrm{of}\;\mathrm{SSF}\;\mathrm{before}\;\mathrm{extraction}\;\left(g\right)\;-\;\mathrm{weight}\;\mathrm{of}\;\mathrm{SSF}\;\mathrm{after}\;\mathrm{extraction}\;(g)}{\mathrm{weight}\;\mathrm{of}\;\mathrm{SSF}\;\mathrm{before}\;\mathrm{extraction}\;\left(g\right)}\;$$

(b) Lignin content

Lignin content was determined following the TAPPI T 222 om-02 standard method [26]. An extractive-free sample (1 g) was transferred to a 100 mL beaker, to which 15 mL of chilled sulfuric acid was added. The beaker was covered with a watch glass and maintained in an ice bath at approximately 20 °C, with stirring performed every 15 min for a duration of 2 h. Subsequently, the reaction mixture was transferred to a 1000 mL round-bottom flask containing 400 mL of distilled water. The beaker was rinsed with 3% sulfuric acid, and the total volume was adjusted to 575 mL, using distilled water. The suspension was then boiled for 4 h while maintaining a constant liquid volume. After standing overnight, the mixture was filtered through a filtering crucible, thoroughly washed with hot distilled water, and dried at 105 °C for 6 h. The lignin content was calculated according to Equation (4).

(4) $$\mathrm{Lignin}\;(\%)=\frac{\mathrm{Lignin}\;(g)}{\mathrm{Extractive}\text{-}\mathrm{free}\;\mathrm{wood}\;(g)+\mathrm{Extractive}\;(g)}\times 100$$

#### 2.4.2. The Chemical Structure of the Bio-Hybrids with Levodopa

Fourier transform infrared (FTIR) spectroscopy (FT/IR-4700, Jasco, Tokyo, Japan) was used to evaluate interactions among formulation components. The samples were analyzed at a resolution of 4 cm^−1^ in transmittance mode in the range of 500–4000 cm^−1^.

#### 2.4.3. Printability Evaluation

Printability was determined by weight, volume, height, and diameter variation. Five samples from each printed formulation were weighed on an analytical balance (LAB 214I, Adam Equipment, Milton Keyens, UK) to obtain the average weight. All formulations contained a high proportion of PVA, and thus, the printed weight was expected to be close to the printed object, which was composed of 100% PVA (PPVA). Formulations were accepted if their average weight was in the range of PPVA ± 5%. The height (h) and diameter (2r) of the prototype were measured by a vernier caliper, and volume (V) was calculated by Equation (5).

V = πr^2^h (5)

The standard deviation (SD), relative standard deviation (RSD), and relative error (%) of the height and diameter were calculated following Equation (6). The acceptance criteria were set as RSD ≤ 5% for unit height and diameter [27].

(6) $$\mathrm{Relative}\;\mathrm{Error}\;(\%)=\frac{\left|\mathrm{Measured}\;\mathrm{value}-\mathrm{Real}\;\mathrm{value}\right|}{\mathrm{Real}\;\mathrm{value}}\times 100$$

where

The measured value is the value obtained from measuring.
The real value is the designed value.

#### 2.4.4. Printlet Characterization

(a) Morphology

Morphological characteristics were observed by scanning electron microscopy (SEM; Tescan Clara™, Tescan Essence Software, Brno, Czech Republic), operated at 15 kV under high vacuum. Samples were fixed on aluminum stubs using double-sided carbon tape, sputter-coated with gold of 5 nm thickness, and imaged at 50× magnification.

(b) Crystallinity

The method to evaluate crystallinity using an X-ray diffractometer (XRD) was adapted from [28]. Detailed experimental conditions are provided in the Supplementary Materials (Table S1). The crystallinity was compared using the crystallinity index (CrI), which is determined from I_am_ as the intensity of the amorphous form and I_002_ as the intensity of the crystalline form, following Equation (7) [29].

(7) $$\mathrm{CrI}=\frac{\left(I_{002}-I_{\mathrm{am}}\right)}{I_{002}}\times 100$$

where

CrI is the crystallinity index.
I_am_ is the intensity of diffraction at 18°.
I_002_ is the maximum intensity of the 002 lattice diffraction.

(c) Thermal properties

The method for studying thermal properties using Differential Scanning Calorimetry (DSC) was adapted from [30]. Detailed experimental conditions are provided in the Supplementary Materials (Table S1).

(d) Shape-changing evaluation

The 3D-printed objects were printed, and their shape was fixed using heat over the glass transition temperature of PVA at 100 °C, and an external force was applied by placing a calibrated weight of 50 g on the 3D-printed objects for 10 s. The temperature of the printlets was cooled down to the room temperature (25 ± 2 °C), then the printlets were soaked in simulated gastric solution, 1000 mL of 0.01 N HCl maintained at 37 ± 2 °C. The opening angle (°) at 20 min was compared to the beginning using ImageJ software version 1.53. The %Recovery was calculated following Equation (8) [31].

(8) $$R_{r}=\frac{\left(\theta_{f}-\theta_{0}\right)}{\left(\theta_{t}-\theta_{0}\right)}\times 100$$

where

R_r_ is the shape recovery ratio.
$\theta_{t}$ is the angle after recovery.
$\theta_{0}$ is the angle after fixing.
$\theta_{f}$ is the angle at time.

(e) Physical and mechanical properties

The thickness of the printlets was measured by a vernier caliper. The method to evaluate the mechanical properties of the printlets using a texture analyzer was described in [12]. Detailed experimental conditions are provided in the Supplementary Materials (Table S1). All measurements were conducted in six replicates. The puncture strength was calculated by following Equation (9).

(9) $$\mathrm{Puncture}\;\mathrm{strength}\;(N/{\mathrm{mm}}^{2})=\frac{F_{\max}}{A}$$

where

F_max_ is the maximum force (N).
A is the contact surface area (mm^2^).

(f) Drug loading content

The method to determine levodopa content using HPLC was adapted from [32]. Detailed experimental conditions are provided in the Supplementary Materials (Table S1).

(g) Drug-releasing properties

The method to determine drug releasing properties of levodopa using HPLC was adapted from [32]. Detailed experimental conditions are provided in the Supplementary Materials (Table S1).

The release data were fitted with kinetic models, for instance, the zero-order, first-order, Hixon–Crowell, Korsmeyer–Peppas, and Higuchi models [33], described in Equations (10)–(13):

Zero-order model:

Q_t_ = Q_0_ + K_z_t (10)

First-order model:

InQ_t_ = InQ_0_ + K_f_t (11)

Higuchi model:

Q_t_ = K_H_ t^1/2^ (12)

Korsmeyer-Peppas model:

Q_t_/Q_∞_ = K_KP_t^n^ (13)

where Q_0_, Q_t_, and Q_∞_ are the initial amount of levodopa before release, the amount of levodopa released at time t, and the total amount of drug dissolved, respectively. K_z_, K_f_, K_H_, and K_KP_ are the drug-release rate constants for the zero-order, first-order, Higuchi model, and Korsmeyer–Peppas model, respectively.

### 2.5. Statistical Analysis

In this study, results are expressed as mean ± standard deviation (SD). Statistical significance (*p* < 0.05) was determined using a one-way ANOVA performed with SPSS software, version 17.0 (IBM Corp., Armonk, NY, USA).

## 3. Results and Discussion

### 3.1. Chemical Composition of Short Fibers

Table 3 presents the chemical composition of the short fibers. The extraction yield of CSF and PSF was 37.84 ± 3.02 and 35.17 ± 2.68%, respectively. The CSF contained a lower amount of holocellulose (46.06 ± 1.79%) and a higher amount of lignin (5.40 ± 0.19%) than PSF (56.8 ± 1.14% of holocellulose and 4.43 ± 0.24% of lignin). The previous study reported that chemical treatment with NaOH was effective in eradicating lignin [34]. In addition, delignification using NaOH exhibited better performance at high temperatures [35]. Boonterm et al. [36] reported that increasing the NaOH concentration during rice straw pulping led to a reduction in fiber yield, as well as the production of smaller fibers with decreased diameter and length.

### 3.2. The Chemical Structure of the Bio-Hybrids with Levodopa

**Figure 4:**
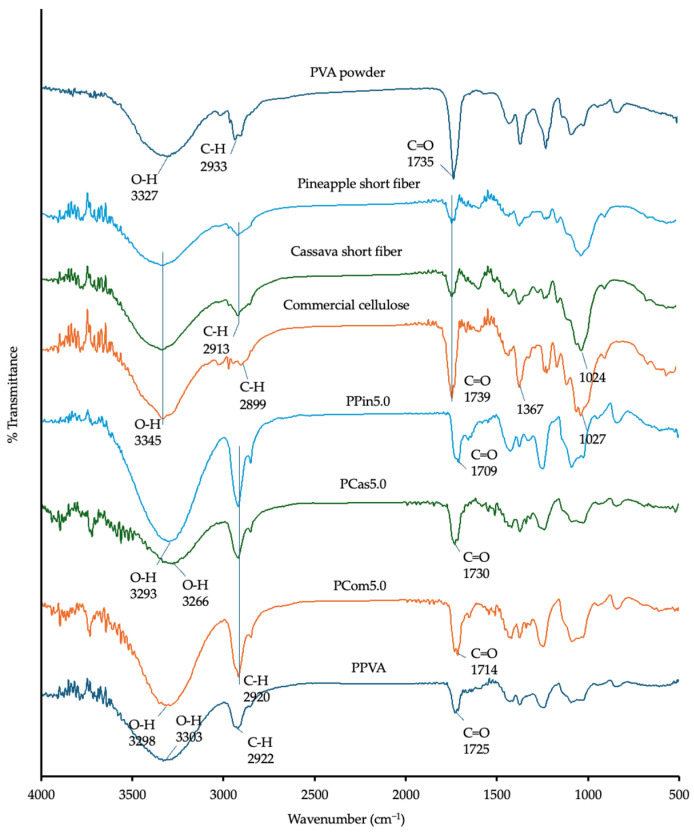
FTIR spectra of raw materials and powder melt extruded PVA-based formulations containing cellulose powder, cassava short fiber, and pineapple short fiber.

**Figure 5:**
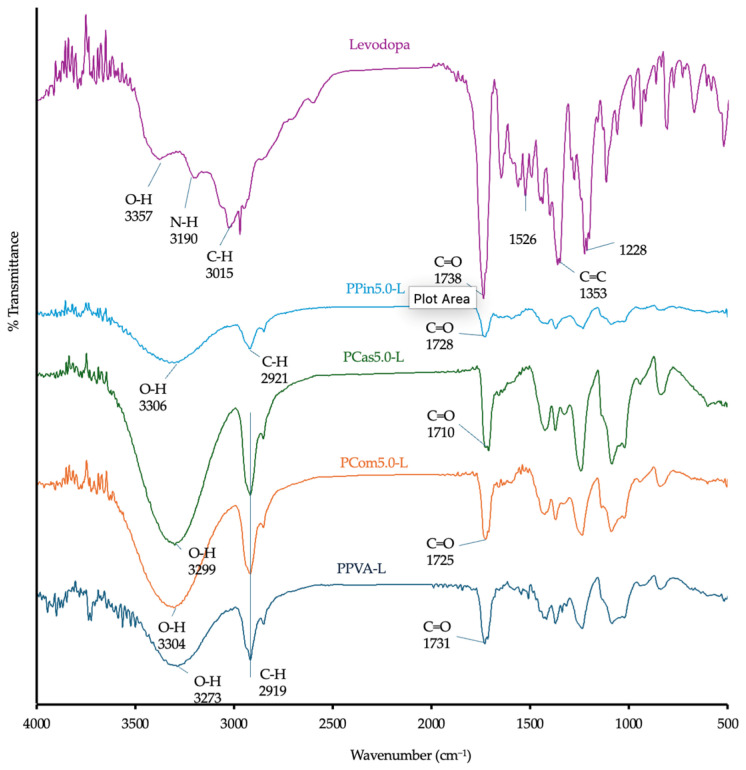
FTIR spectra of levodopa and powder melt extruded PVA-based formulations containing levodopa with cellulose powder, cassava short fiber, and pineapple short fiber.

The chemical interaction between levodopa and the polymers was evaluated using Fourier transform infrared (FTIR) spectroscopy. The FTIR spectrum of PVA exhibited a broad –OH band at ~3327 cm^−1^ and a C–H stretching band from alkyl groups at ~2933 cm^−1^ [37]. The cellulose or short fibers displayed a broad –OH band at ~3345 cm^−1^, C–H stretching bands at 2899 and 2913 cm^−1^, and a C=O stretching band around 1739 cm^−1^, as shown in Figure 4 [38]. The spectrum of levodopa exhibited an –OH stretching vibration at ~3357 cm^−1^, an –NH_2_ band at ~3190 cm^−1^, and a C=O stretching band at ~1738 cm^−1^, as shown in Figure 5 [39].

The FTIR analysis of the printed formulations revealed significant shifts in several characteristic peaks when compared with the spectra of the individual components. In the printlets containing PVA, cellulose, and levodopa, the hydroxyl (–OH) stretching bands originally observed at ~3327–3357 cm^−1^ shifted to around 3300 cm^−1^ and exhibited noticeable band broadening. This downward shift and broadening indicate the formation of hydrogen bonds between the –OH groups of PVA and cellulose (or short fibers) and the –OH and –NH_2_ groups of levodopa. Such hydrogen-bonding interactions weaken the O–H stretching vibrations, resulting in the observed shift to lower wavenumbers. Previous studies have reported that hydrogen bonding and high polymer miscibility are key factors in stabilizing the amorphous state of ASD [40]. In Figure 5, the printlets exhibited broader and more intense –OH stretching peaks compared with pure levodopa, attributable to the formation of ASDs, as confirmed by XRD diffraction pattern in Figure 9. Similarly, the carbonyl (C=O) stretching band of levodopa at ~1738 cm^−1^ shifted to lower frequencies (~1710–1730 cm^−1^) in the printlets (Figure 5). This shift suggests that the C=O groups also participated in hydrogen bonding with the hydroxyl groups of PVA and cellulose or short fibers. This observation is consistent with the previous study, reporting a pronounced shift in the C=O stretching band due to intermolecular interactions between the carbonyl group of levodopa and the hydroxyl groups of a polymeric excipient [41].

The formation of ASDs during the printing process, which can transform levodopa from a crystalline to an amorphous state, also influences the positions of the –OH and C=O bands [41]. These spectral changes provide further evidence of molecular interactions between levodopa and the polymeric components (PVA and cellulose or short fiber).

### 3.3. Printing Prototype and Formulation Investigation

Printability was determined based on two criteria: the %RSD of height, diameter, volume, and weight, and the percentage deviation in the weight of each formulation. The acceptance criteria were set at %RSD < 5%. As shown in Table 3, the RSD values for height, diameter, and volume of all formulations were below 5%. However, the RSD of weight for formulation PCom7.5 was 10.34%, which exceeded the acceptance criteria. Therefore, this formulation exhibited improper printability. Formulations consisting of CP, CSF, and PSF showed good particle distribution and particle sizes smaller than the nozzle diameter of 0.8 mm (800 µm) (Figure S1), whereas formulations containing DSF and HSF exhibited agglomeration with particle sizes of approximately 900 µm, which resulted in an inability to print (Figure S1).

An increasing cellulose content enhances the structural strength [42]. It might improve printability and geometric characteristics of formulations. Conversely, low cellulose concentration influences the three-dimensional structure, which lacks sufficient mechanical strength [43]. Previous studies have found that the addition of an appropriate amount of cellulose increases the mechanical strength of PVA 3D-printed samples [44]. However, excessive cellulose concentration could obstruct the material’s flow through the printing nozzle and may lead to deterioration in printing performance [43].

In addition, the percentage deviation in the weight of each formulation was compared with the PVA-based printlet without levodopa. As shown in Table 4, the weight of formulations containing 2.5% or 7.5% of cellulose additives differed by more than 5% from the control (PVA-based printlet without levodopa: 95–105%). These formulations also exhibited inappropriate printability.

### 3.4. Characterizations of the Printlets

#### 3.4.1. 3D Printing of the Designed Printlet and Morphology

**Figure 6:**
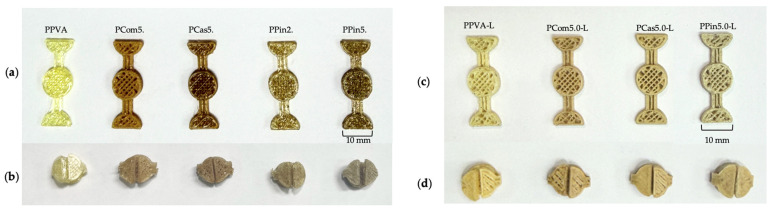
Images of printlets without levodopa: PPVA, PCom5.0, PCas5.0, PPin2.5, and PPin5.0 (**a**) before and (**b**) after fixing the shape, and images of printlets containing levodopa: PPVA-L, PCom5.0-L, PCas5.0-L, and PPin5.0-L (**c**) before and (**d**) after fixing the shape.

**Figure 7:**
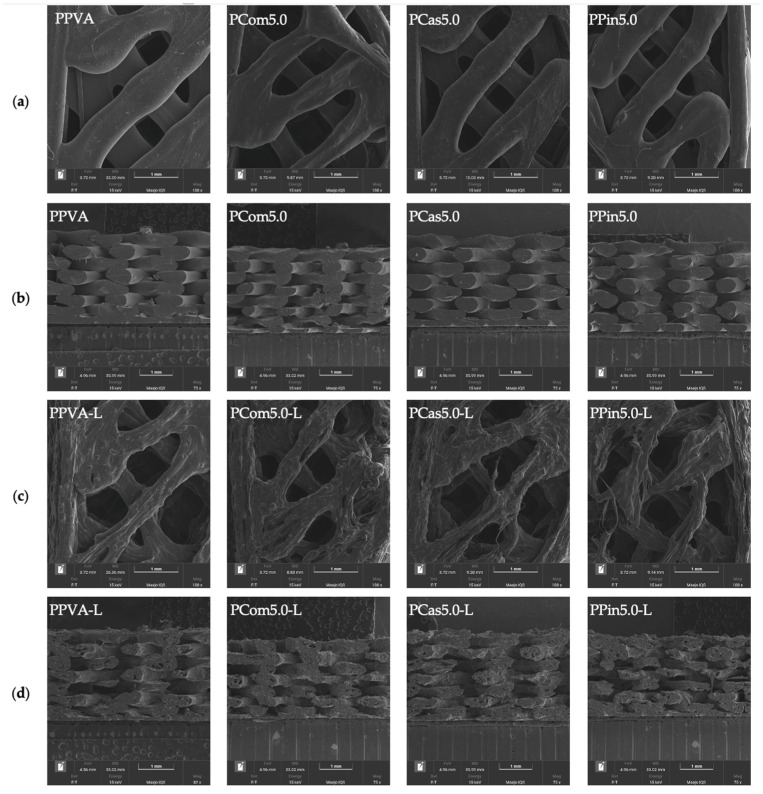
SEM images of printlets without levodopa: PPVA, PCom5.0, PCas5.0, and PPin5.0 at 100× magnification (front view; (**a**)) and 75× magnification (cross sectional view; (**b**)), and SEM images of printlets containing levodopa: PPVA-L, PCom5.0-L, PCas5.0-L, and PPin5.0-L at 100× magnification (front view; (**c**)) and 75× magnification (cross sectional view; (**d**)).

As shown in Figure 6a, all formulations were completely printed according to the design. Each printlet formulation exhibited a different color. Cellulose or short fibers exhibited variations in color due to differences in internal composition, such as lignin [45]. Figure 7a shows that the printlets exhibited relatively smooth surfaces without visible defects, such as tearing or structural damage. However, build lines were still noticeable at the interlayer. Figure 7b (cross-sectional view) shows that cellulose or short fibers were uniformly dispersed throughout the matrix without agglomeration, resulting in a homogeneous structure of the printed objects, and no obvious pores were found in the printlet without levodopa.

When levodopa was loaded in formulations, as shown in Figure 6b, all formulations containing levodopa were successfully printed according to the design. Post-printing quality was evaluated based on the thickness of the printed printlets. The average thickness of PPVA-L, PCom5.0-L, PCas5.0-L, and PPin5.0-L was 2.94 ± 0.04, 2.94 ± 0.03, 2.91 ± 0.05, and 2.96 ± 0.12 mm, respectively, which were close to the designed structure. This thickness can indicate that the printing process exhibited good printability quality after printing, related to path discontinuity, severe stringing, under-extrusion, or layer adhesion. Moreover, those printlets were not measured immediately after printing, but after a short period. Therefore, the printed printlets maintained dimensional stability over time.

Figure 7c shows that the drug-loaded formulations exhibited rougher surfaces compared to the drug-free formulation. In Figure 7d, the cross-sectional view revealed incomplete interlayer adhesion and process-induced pores in all formulations. The pores occurred from the dispersion of the drug in the polymer’s matrix [46]. SEM micrographs also showed that levodopa dispersed homogenously throughout the printlet matrix without aggregation (Figure 7c,d). The printlet from the pineapple short fiber formulation showed noticeable tearing defects. It indicated that levodopa loading affected printability. Specifically, at printing temperature, levodopa was not completely melted but dispersed in melted PVA. This may affect flow behavior. Moreover, the drug loading could also influence the stiffness and consistency of the printlets [47].

#### 3.4.2. Crystallinity

**Figure 8:**
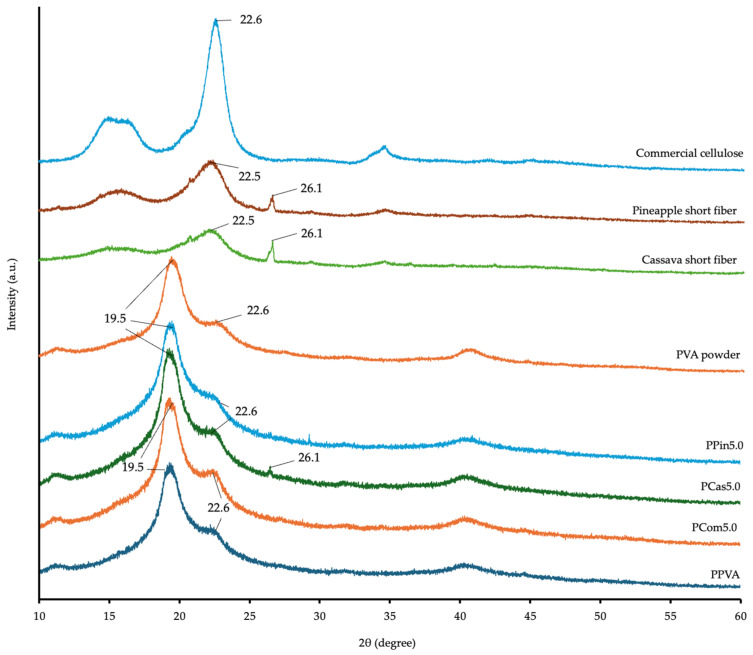
XRD diffraction pattern of raw materials and powder melt extruded PVA-based formulations containing cellulose powder, cassava short fiber, and pineapple short fiber.

Figure 8 shows the crystalline patterns at various positions in the raw materials. Cellulose exhibited a characteristic peak at 22.6°, which was similar to those of pineapple short fiber and cassava short fiber, showing peaks at 22.5° and 22.6° [48]. PVA powder shows diffraction peaks at 19.5° and 22.6° [12]. After printing the formulations without levodopa through PME, the characteristic peaks of cellulose or short fibers were masked, resulting in diffraction patterns partially similar to PVA.

**Figure 9:**
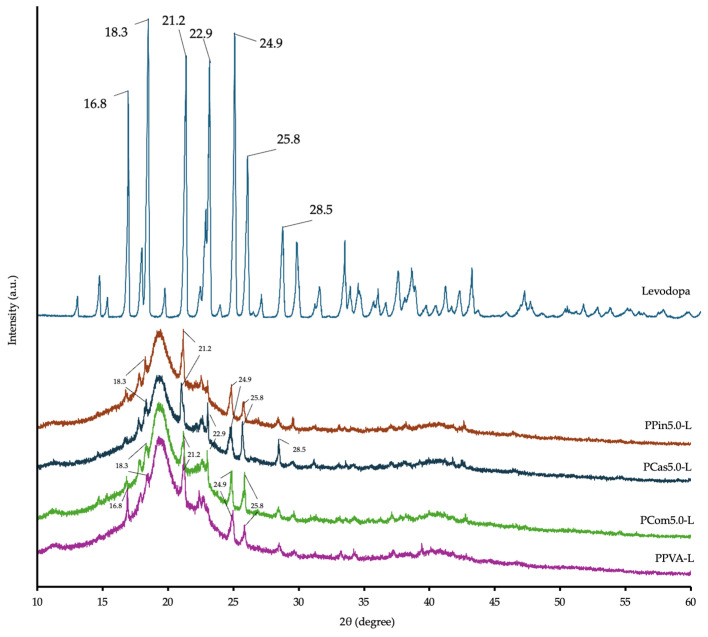
XRD diffraction pattern of levodopa and powder melt extruded PVA-based formulations containing levodopa with PVA, cellulose powder, cassava short fiber, and pineapple short fiber.

As shown in Figure 9, levodopa shows crystallinity peaks at approximately 18.3°, 21.2°, 22.9°, 24.9°, and 25.8° [49]. The results revealed that the peaks of all four formulations containing the levodopa though PME printing as PPVA-L, PCom5.0-L, PCas5.0-L, and PPin5.0-L. The peak of the printed formulation showed lower intensities or reduced crystallinity compared with pure levodopa. This result indicated amorphous drug dispersion within the formulations. The PVA can immobilize drug molecules and reduce their molecular mobility through the formation of ASD. After printing all levodopa-loaded formulations, the crystallinity in levodopa was markedly reduced and transformed to a semi-amorphous state [50]. A previous study also reported reduced crystallinity in dronedarone with ASDs [51].

According to Segal et al. [52], the CrI can be approximately evaluated by comparing the degree of crystallinity among the different formulations. As shown in Table 5, the CrI values varied among the formulations, with PPin5.0-L exhibiting the highest crystallinity (30.08), followed by PPVA-L and PCom5.0-L (27.53 and 27.38, respectively), while PCas5.0-L showed the lowest value (17.36). The results show that cassava short fiber was the most effective in reducing crystallinity, followed by cellulose powder, while pineapple short fiber exhibited the least effect. Furthermore, the printlets containing levodopa exhibited a marked reduction in crystallinity compared to the levodopa powder, which was attributable to ASDs [53].

These results show that different types of cellulose promote ASD and reduce crystallinity to different extents. According to Luebbert et al. [54], differences in cellulose molecular weight affect the formation of ASD and the crystallinity in the resulting materials.

#### 3.4.3. Thermal Properties

**Figure 10:**
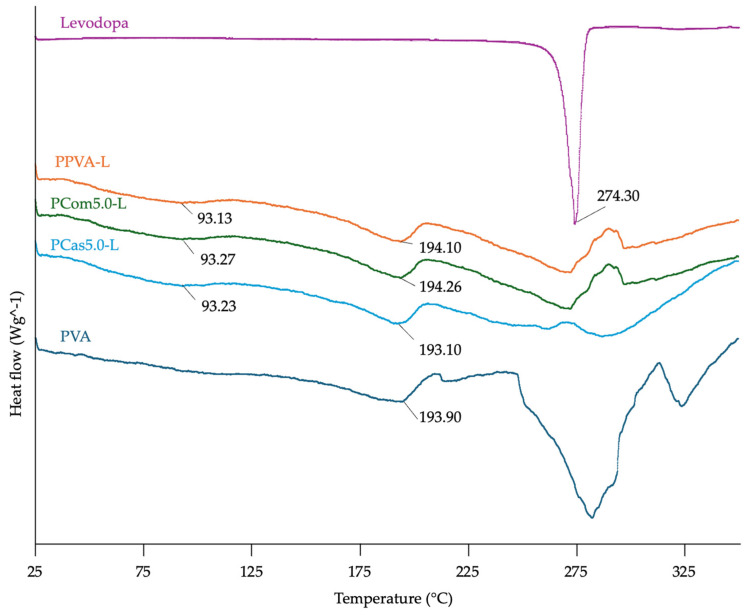
DSC thermograms of PVA powder, levodopa, and powder melt extruded formulations containing levodopa with PVA, cellulose powder, and cassava short fiber.

As shown in Figure 10, the results show that levodopa has a melting point (T_m_) of 274.3 °C [55], while PVA exhibits a melting point of 193.90 °C. These findings are consistent with previous studies showing that PVA has a melting peak around 190 °C [56]. The printlets containing PVA show thermograms similar to PVA. Moreover, the printlets exhibited a glass transition temperature (T_g_) at approximately 93 °C [57]. Based on thermal properties, the shape-changing evaluation was designed by heating the printlets to a temperature of T_g_ + 10 °C. When the material is heated above T_g_, it becomes flexible enough to be formed into the desired shape [58]. The thermograms showed that the melting peak of levodopa at 274.30 °C disappeared after printing. The disappearance of this melting peak indicates that the ASDs may have transformed levodopa from the crystalline state into an amorphous form, thereby reducing the crystallinity in the drug [59].

#### 3.4.4. Shape-Changing Evaluation of Printlets

Table 6 shows that the printlets of all formulations gradually expanded over time. At 15 min, the PPVA, PPin2.5, and PPVA-L formulations dissolved and could not maintain their designed shapes (Table S2). Moreover, a comparison between printlets with the same short fiber, PPin2.5 and PPin5.0, revealed that PPin5.0 exhibited a high % recovery and was able to maintain shape at 15 min. Consequently, PPin2.5 was dissolved at 15 min. At 20 min, the PCom5.0, PCas5.0, and PPin5.0 formulations dissolved and were unable to maintain their designed (Table S2).

A previous study reported that the addition of cellulose decreased the degradation of PVA hydrogels in water due to hydrogen bonding between PVA and cellulose [60]. (PCom5.0-L and PCas5.0-L) exhibited no significant difference in % recovery at 20 min (85.37 ± 13.02% and 87.33 ± 21.95%, respectively) but demonstrated significantly higher % recovery than (PPin5.0-L) (39.05 ± 23.55%). Moreover, the PPin5.0-L printlet had a torn appearance, observed in the SEM micrograph (Figure 7c), which resulted in poorer shape recovery performance compared with the other formulations [61]. The formulation containing cellulose or short fibers maintained its designed shapes for a longer period than the formulation without cellulose or short fibers because the cellulose or short fibers increased the mechanical strength and delayed overall solubility of the printlets [62]. Formulations that maintain their structure and exhibit effective expansion over time can undergo shape transformation to a larger size, thereby prolonging gastric residence time and enhancing the efficiency of controlled drug release.

#### 3.4.5. Physical and Mechanical Properties

The mechanical properties of the printlets are presented in Table 7. The puncture strength reflects the hardness of the material, relating to the structural stability of the printlets. PPVA-L exhibited the highest puncture strength (41.10 ± 3.79 N/mm^2^) among all formulations. Upon the incorporation of cellulose powder and cassava short fiber, the puncture strength significantly decreased to 31.49 ± 3.50 and 30.82 ± 3.90 N/mm^2^, respectively. A previous study reported that the addition of cellulose reduced the tensile strength of filaments used for 3D printing [63]. Moreover, the presence of cellulose and short fibers may interfere with the interactions between PVA and levodopa, leading to a reduction in puncture strength. When cellulose powder and cassava short fiber were incorporated, the puncture strength significantly decreased to 31.49 ± 3.50 and 30.82 ± 3.90 N/mm^2^, respectively. The levodopa-loaded printlet with 5% pineapple short fiber (PPin5.0-L) exhibited the lowest puncture strength (21.74 ± 3.16 N/mm^2^), indicating inferior structural strength compared with the other formulations. This reduced strength suggests that PPin5.0-L is more prone to tearing, as observed in the SEM micrograph (Figure 7c), and exhibited poorer shape recovery performance due to faster degradation in the test medium.

#### 3.4.6. Drug Loading Content

The levodopa contents of PPVA-L, PCom5.0-L, and PCas5.0-L were 25.11 ± 1.03, 25.40 ± 1.35, and 24.82 ± 0.89 mg, or 100.43 ± 4.1, 101.59 ± 5.38, and 99.27 ± 3.56, respectively (Table 8). The levodopa content is not significantly different between each formulation. The high drug content (around 100%) and low standard deviation are related to the consistency of the printing process and homogeneous dispersion of the drug in the polymer’s matrix. In addition, the drug loading content of PPVA-L, PCom5.0-L, and PCas5.0-L complies with the USP specification for levodopa extended release tablet that specifies that the drug content in a drug product must in ranging of 90–110% [64].

#### 3.4.7. Drug Releasing Properties

**Figure 11:**
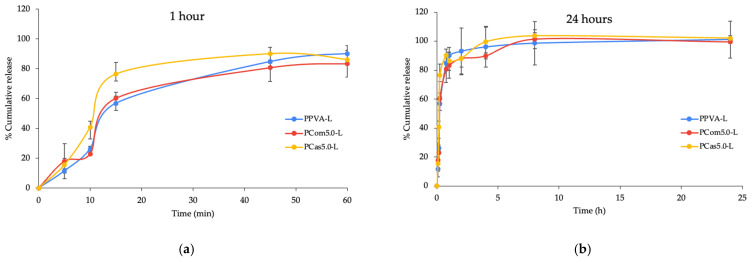
Drug release of printlet containing levodopa with cellulose powder or cassava in the initial 1 h (**a**) and over 24 h (**b**).

Levodopa powder completely dissolved within 1 min, indicating immediate drug release. As shown in Figure 11a,b, PPVA-L, PCom5.0-L, and PCas5.0-L showed a gradual release of levodopa over the first 15 min. During the first 45 min, the levodopa-loaded printlet with cassava short fiber (PCas5.0-L) exhibited a faster drug release compared with the other formulations (Figure 11a). After 60 min, the drug release was approximately 90% and gradually increased to around 100% at 8 h in all formulations (Figure 11b). The levodopa-loaded printlets prepared by PME demonstrated the enhanced solubility of levodopa due to the formation of ASDs. The XRD analysis further supports this mechanism, as PCas5.0-L exhibited the lowest crystallinity index among all formulations (CrI = 17.36), indicating a more amorphous matrix.

The release profile of levodopa from the printlets was fixed using different kinetic models, including zero-order, first-order, Higuchi, and Korsmeyer–Peppas models. The correlation coefficient (R^2^) and the corresponding release rate constants obtained from these models are presented in Table 9. The results indicate that the PPVA-L formulation best fits the Higuchi model, while both PCom5.0-L and PCas5.0-L fit the Korsmeyer–Peppas model, as evidenced by their highest R^2^ values. The good agreement of PPVA-L with the Higuchi model suggests that its drug release is primarily governed by diffusion through the matrix. In contrast, the Korsmeyer–Peppas model provides further insight into the release mechanisms of the PCom5.0-L and PCas5.0-L formulations based on their release exponent (n) values shown in Table 9.

For PCas5.0-L, the n value was greater than 1, indicating a Super Case II transport mechanism (Table 9) [65]. This suggests that drug release is dominated by polymer relaxation and pronounced swelling [66,67]. This release may be attributed to the high water uptake of cassava short fibers, as shown in Table S2. Furthermore, the highly porous structure of PCas5.0-L (Figure 7c,d) enhances water uptake, leading to support swelling behavior. A previous study reported that PVA-based hydrogels with highly porous structures exhibit high water absorption and rapid swelling rates [68]. The increased amorphous content of cassava short fiber enhances water uptake and accelerates polymer chain relaxation, which is consistent with the observed Super Case II transport behavior. Meanwhile, levodopa-loaded printlets containing only PVA or cellulose powder exhibit an n value within 0.45 < n < 0.89, which corresponds to non-Fickian diffusion (Table 9) [66]. The Korsmeyer–Peppas analysis revealed that different release mechanisms exist among the formulations. PCas5.0-L, which has a super Case II transport mechanism, exhibited a faster initial drug release than PPVA-L and PCom5.0-L.

## 4. Conclusions

This study demonstrates a novel application of the use of cellulose and short fibers for PME processing by demonstrating shape-changing levodopa delivery. The formulation was selected based on the type and concentration of cellulose or short fiber, considering their printability and printlet weight. Printlets consisting of cellulose powder, cassava short fiber, and pineapple short fiber were successfully produced through PME according to the designed geometry. The shape-changing behavior of the selected printlets, including the levodopa-loaded printlet, showed that the incorporation of cellulose and levodopa enhanced the ability to maintain their structure during shape transformation in simulated gastric fluid. Among the formulations, Cassava short fiber provided the greatest ability to support shape expansion, contributing to swelling-induced deformation while maintaining structure. XRD analysis revealed that PME reduced crystallinity through the formation of ASDs and exhibited the greatest crystallinity reduction. Furthermore, FTIR spectra indicated molecular-level interactions between levodopa and the PVA-based system to confirm ASDs formation within the formulations. The drug release profile showed that the levodopa-loaded printlet with cassava short fiber (5.0%) exhibited higher initial release than the other formulations due to rapid swelling within the first 20 min. This result was supported by the lowest crystallinity and the presence of a super Case II transport mechanism. Overall, these findings highlight the functional role of cellulose powder and cassava short fiber as sustainable additives in PME processing. This work extends the application of PME by integrating agricultural waste-derived fibers into shape-adaptive pharmaceutical dosage forms. Future studies may require adjustments in drug dosage or further optimization of the manufacturing process to improve practical applicability.

## Figures and Tables

**Table 1:** Material composition of PVA, cellulose powder, and short fibers in each formulation.

Formulations	Material Composition (% w/w)					
	PVA	CP	CSF	PSF	DSF	HSF
PPVA	100.0	-	-	-	-	-
PCom2.5	97.5	2.5	-	-	-	-
PCom5.0	95.0	5.0	-	-	-	-
PCom7.5	92.5	7.5	-	-	-	-
PCas2.5	97.5	-	2.5	-	-	-
PCas5.0	95.0	-	5.0	-	-	-
PCas7.5	92.5	-	7.5	-	-	-
PPin2.5	97.5	-	-	2.5	-	-
PPin5.0	95.0	-	-	5.0	-	-
PPin7.5	92.5	-	-	7.5	-	-
PDu2.5	97.5	-	-	-	2.5	-
PDu5.0	95.0	-	-	-	5.0	-
PDu7.5	92.5	-	-	-	7.5	-
PHe2.5	97.5	-	-	-	-	2.5
PHe5.0	95.0	-	-	-	-	5.0
Phe7.5	92.5	-	-	-	-	7.5

**Table 2:** Material composition of the selected formulations containing levodopa.

Formulations	Material Composition (% w/w)				
	PVA	CP	CSF	PSF	Levodopa
PPVA-L	90.0	-	-	-	10.0
PCom5.0-L	85.0	5.0	-	-	10.0
PCas5.0-L	85.0	-	5.0	-	10.0
PPin2.5-L	87.5	-	-	2.5	10.0
PPin5.0-L	85.0	-	-	5.0	10.0

**Table 3:** Chemical composition of short fibers.

Sample	Yield (%)	Holocellulose (%)	Lignin (%)
CSF	37.84 ± 3.02 ^a^	46.06 ± 1.79 ^a^	5.40 ± 0.19 ^a^
PSF	35.17 ± 2.68 ^a^	56.8 ± 1.14 ^b^	4.43 ± 0.24 ^b^

Different superscript letters in each column indicate significant difference (*p* < 0.05).

**Table 4:** Height and diameter of prototype in each formulation.

Formulation	Height (mm) ± SD	%RSD Height	Relative Error of Height ± SD	Diameter (mm) ± SD	%RSD Diameter	Relative Error of Diameter ± SD	volume (cm^3^) ± SD	%RSD Volume	Relative Error of Volume ± SD	Weight (mg) ± SD	%RSD Weight	% Deviation in Weight
PPVA	5.77 ± 0.22 ^a^	3.81	7.00 ± 3.71	9.67 ± 0.02 ^a^	0.21	3.28 ± 0.16	423.91 ± 15.25 ^ab^	1.02	2.53 ± 2.22	268.93 ± 3.90 ^abc^	1.45	Control
PCom2.5	5.75 ± 0.08 ^ab^	1.39	6.44 ± 1.41	9.68 ± 0.07 ^a^	0.72	3.20 ± 0.73	422.05 ± 11.24 ^ab^	0.63	2.11 ± 1.33	251.30 ± 12.59 ^b^	5.01	93.44
PCom5.0	5.71 ± 0.05 ^ab^	0.88	5.67 ± 0.83	9.81 ± 0.03 ^b^	0.31	1.92 ± 0.33	431.11 ± 4.25 ^a^	0.61	1.65 ± 1.00	281.97 ± 6.79 ^ac^	2.41	104.85
PCom7.5	5.64 ± 0.04 ^b^	0.71	4.41 ± 0.69	9.66 ± 0.09 ^a^	0.93	3.44 ± 0.89	412.90 ± 8.70 ^bc^	0.71	2.71 ± 1.94	277.20 ± 28.67 ^ac^	10.34	103.07
PCas2.5	5.33 ± 0.09 ^cd^	1.69	1.56 ± 1.32	9.61 ± 0.03 ^a^	0.31	3.92 ± 0.33	386.32 ± 8.20 ^d^	0.22	8.91 ± 1.93	287.13 ± 8.64 ^c^	3.01	106.77
PCas5.0	5.39 ± 0.05 ^c^	0.93	0.70 ± 0.42	9.64 ± 0.13 ^a^	1.35	3.62 ± 1.26	393.62 ± 12.41 ^d^	0.41	7.19 ± 2.93	281.97 ± 6.79 ^ac^	2.41	104.85
PCas7.5	5.36 ± 0.05 ^c^	0.93	0.89 ± 0.77	9.61 ± 0.05 ^a^	0.52	3.92 ± 0.52	388.32 ± 4.16 ^d^	0.12	8.44 ± 0.98	368.90 ± 10.49 ^d^	2.84	137.17
PPin2.5	5.33 ± 0.08 ^cd^	1.50	1.89 ± 0.46	9.88 ± 0.06 ^bc^	0.61	1.22 ± 0.57	408.22 ± 10.52 ^ce^	0.58	3.86 ± 2.25	276.50 ± 4.39 ^ac^	1.59	102.81
PPin5.0	5.33 ± 0.03 ^cd^	0.56	1.26 ± 0.56	9.94 ± 0.04 ^cd^	0.40	0.62 ± 0.38	413.62 ± 5.41 ^bef^	1.02	2.47 ± 1.28	266.67 ± 1.88 ^b^	0.71	99.16
PPin7.5	5.23 ± 0.10 ^d^	1.91	3.11 ± 1.93	9.97 ± 0.05 ^d^	0.50	0.42 ± 0.36	408.10 ± 6.41 ^cf^	0.63	3.78 ± 1.51	254.73 ± 4.22 ^b^	1.66	94.72

Different superscript letters in each column indicate significant difference (*p* < 0.05).

**Table 5:** The relative degree of crystallinity in each levodopa-loaded formulation.

Formulation	I_002_	I_am_	CrI
Levodopa	13,014.00	1008.00	92.25
PPVA-L	3167.00	2295.00	27.53
PCom5.0-L	2926.00	2125.00	27.38
PCas5.0-L	2201.00	1819.00	17.36
PPin5.0-L	2676.00	1871.00	30.08

**Table 6:** %Recovery shape of powder melt extruded PVA-based formulations with and without levodopa, containing cellulose powder, cassava short fiber, and pineapple short fiber, evaluated over time.

Formulation	0 min	5 min	10 min	15 min	20 min
PPVA	0	38.97 ± 21.04 ^a^	52.06 ± 15.31 ^a^	ND	ND
PCom5.0	0	23.7 ± 2.25 ^ab^	47.85 ± 11.92 ^a^	100 ± 0 ^a^	ND
PCas5.0	0	32.08 ± 3.62 ^ac^	45.97 ± 13.13 ^a^	89.56 ± 14.76 ^ab^	ND
PPin2.5	0	31.29 ± 4.41 ^ac^	40.21 ± 9.05 ^a^	ND	ND
PPin5.0	0	45.64 ± 21.75 ^a^	55.49 ± 26.2 ^a^	80.35 ± 3.22 ^ab^	ND
PPVA-L	0	16.81 ± 10.74 ^bc^	24.92 ± 13.25 ^a^	ND	ND
PCom5.0-L	0	9.87 ± 6.93 ^b^	52.02 ± 27.74 ^a^	76.17 ± 21.49 ^ab^	85.37 ± 13.02 ^a^
PCas5.0-L	0	7.74 ± 7.88 ^b^	31.76 ± 20.42 ^a^	55.43 ± 30.45 ^bc^	87.33 ± 21.95 ^a^
PPin5.0-L	0	4.19 ± 2.99 ^b^	25.34 ± 29.43 ^a^	28.78 ± 28.3 ^c^	39.05 ± 23.55 ^b^

Different superscript letters in each column indicate significant difference (*p* < 0.05). ND: not determined.

**Table 7:** Mechanical properties of printlets containing levodopa.

Formulation	Puncture Strength (N/mm^2^)
PPVA-L	41.10 ± 3.79 ^a^
PCom5.0-L	31.49 ± 3.50 ^b^
PCas5.0-L	30.82 ± 3.90 ^b^
PPin5.0-L	21.74 ± 3.16 ^c^

Different superscript letters in each column indicate significant difference (*p* < 0.05).

**Table 8:** Levodopa content in each formulation.

Formulation	Levodopa (mg) ± SD	Levodopa (%) ± SD
PPVA-L	25.11 ± 1.03 ^a^	100.43 ± 4.1 ^a^
PCom5.0-L	25.40 ± 1.35 ^a^	101.59 ± 5.38 ^a^
PCas5.0-L	24.82 ± 0.89 ^a^	99.27 ± 3.56 ^a^

Different superscript letters in each column indicate significant difference (*p* < 0.05).

**Table 9:** Released kinetic profile of printlet containing levodopa with cellulose powder or cassava short fiber in drug release models.

Kinetic Models.	Parameter	Sample		
		PPVA-L	PCom5.0-L	PCas5.0-L
Zero-order	R^2^	0.872	0.817	0.983
	K_0_ (h^−1^)	7.690	10.334	9.096
First-order	R^2^	0.434	0.773	0.402
	K_1_ (h^−1^)	0.482	0.026	0.721
Higuchi	R^2^	0.942	0.941	0.879
	K_H_ (h^1/2^)	91.338	90.135	114.400
Korsmeyer–Peppas	R^2^	0.919	0.941	0.999
	K_KP_ (h^−n^)	94.014	101.487	617.076
	n	0.668	0.637	1.512

## Data Availability

The original contributions presented in this study are included in the article/Supplementary Materials. Further inquiries can be directed to the corresponding author.

## Supplementary Materials

The following supporting information can be downloaded at: https://www.mdpi.com/article/10.3390/polym18030380/s1, Figure S1: Powder appearance of PCom2.5, PCas2.5, PPin2.5, PDu2.5 and PHe2.5 formulations on 4× magnification under a microscope (Eclipse E200, Nikon Corporation, Tokyo, Japan); Table S1: Equipment and experimental parameters of Printlets characterization; Table S2: Shaping changing of PPVA, PCom5.0, PCas5.0, PPin2.5, PPin5.0, PPVA, PCom5.0-L, PCas5.0-L, and PPin5.0-L.

## Abbreviations

The following abbreviations are used in this manuscript:

ASDs	Amorphous solid dispersions
CSF	Cassava short fiber
CP	Cellulose powder
CrI	Crystallinity index
DSF	Durian short fiber
HME	Hot-melt extrusion
HSF	Hemp short fiber
PME	Powder melt extrusion
PSF	Pineapple short fiber
