# Supplementary material for: Additive Manufacturing of Shape-Changing Printlets via Powder-Based Extrusion 3D Printing of Natural Cellulose and Polyvinyl Alcohol
Source: Polymers (Basel). 2026 Jan 30;18(3):380. doi: 10.3390/polym18030380 (PMC12899022; doi:10.3390/polym18030380)
Supplement: Supplementary file 1 [file polymers-18-00380-s001.zip › polymers-4075956-supplementary.pdf]

polymers

MDPI

Supplementary Materials

Additive Manufacturing of Shape-Changing Oral Tablets via Powder-Based Extrusion 3D Printing of Natural Cellulose and Polyvinyl Alcohol

Kasidit Dokhom [1], Pensak Jantrawut [1,2], Pattaraporn Panraksa [1], Suruk Udomsom [3,4], Wirongrong Tongdeesoontorn [5,6], Baramee Chanabodeechalermrung [1], Pornchai Rachtanapun [2,7], and Tanpong Chaiwarit [1,*]

[1] Department of Pharmaceutical Sciences, Faculty of Pharmacy, Chiang Mai University, Chiang Mai 50200, Thailand; kasidit_dokh@cmu.ac.th (K.D.); pensak.j@cmu.ac.th (P.J.); pattaraporn.pan@cmu.ac.th (P.P.); baramee.c@cmu.ac.th (B.C.); tanpong.ch@cmu.ac.th (T.C.)
[2] Center of Excellence in Agro Bio-Circular-Green Industry (Agro BCG), Agro-Industry, Chiang Mai University, Chiang Mai 50100, Thailand
[3] Department of Electrical Engineering, Faculty of Engineering, Chiang Mai University, Chiang Mai, 50200 Thailand; suruk.u@cmu.ac.th (S.U.)
[4] Office of Research Administration, Chiang Mai University, Chiang Mai 50200, Thailand
[5] School of Agro-Industry, Mae Fah Luang University, 333 Moo 1 Tasud, Chiang Rai 57100, Thailand; wirongrong.ton@mfu.ac.th (W.T.)
[6] Research Center of Innovative Food Packaging and Biomaterials Unit, Mae Fah Luang University, 333 Moo 1 Tasud, Chiang Rai 57100, Thailand
[7] Faculty of Agro-Industry, Chiang Mai University, Mae-Hea, Mueang, Chiang Mai 50100, Thailand; pornchai.r@cmu.ac.th (P.R.)
* Correspondence: tanpong.ch@cmu.ac.th; Tel.: +66882-610-254

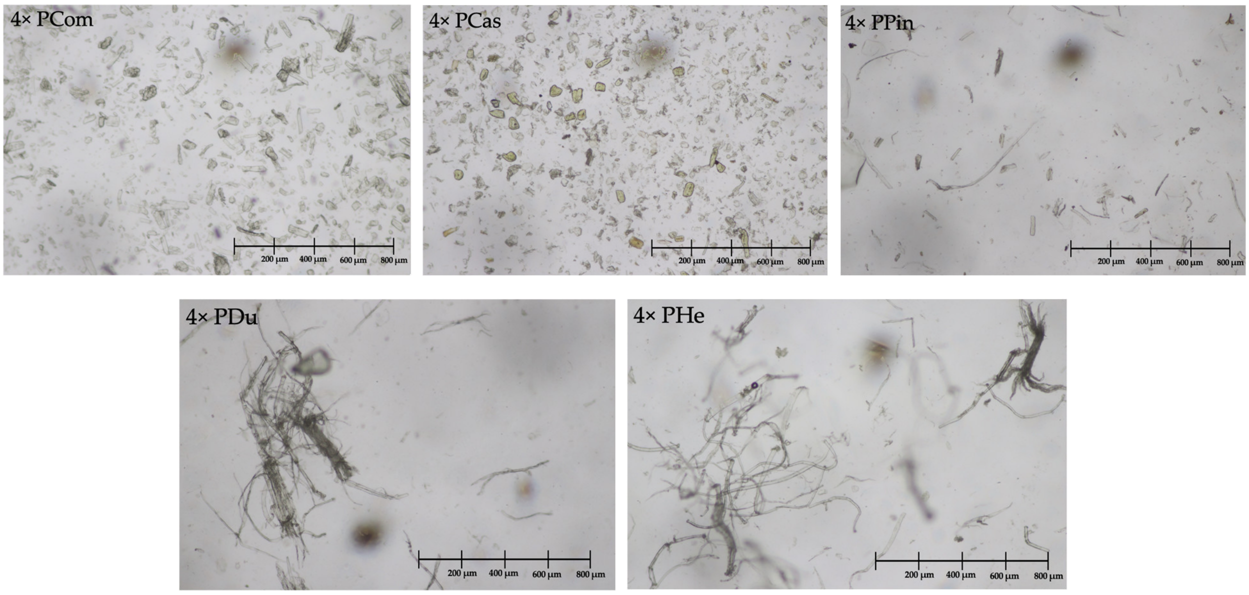

Figure S1. Powder appearance of PCom2.5, PCas2.5, PPin2.5, PDu2.5 and PHe2.5 formulations on 4× magnification under a microscope (Eclipse E200, Nikon Corporation, Tokyo, Japan)

Table S1. Equipment and experimental parameters of Printlets characterization

Section	Equipment	Condition
Morphology	Scanning electron microscopy (SEM; Tescan Clara™, Tescan Essence Software, Brno, Czech Republic)	Morphological characteristic was observed by SEM, operated at 15 kV under high vacuum. Samples were fixed on aluminum stubs using double-sided carbon tape, sputter-coated with gold of 5 nm thickness, and imaged at 50× magnification.
XRD analysis	X-ray diffractometer (Empyrean Series 3; Malvern Panalytical, Malvern, UK)	Crystallinity of samples before and after printing was evaluated by X-ray diffraction (XRD) using an X-ray diffractometer (Empyrean Series 3; Malvern Panalytical, Malvern, UK). Diffraction patterns were collected over 10–60° (2θ) at a scan speed of 10°/min with a step size of 0.01°, operating at 40 kV.
Thermal properties	Differential scanning calorimetry (DSC; Mettler Toledo Stare System, DSC N1 Module, Greifensee, Switzerland)	Samples (2 mg) were sealed in 40-μL platinum pans and analyzed by differential scanning calorimetry (DSC; Mettler Toledo Stare System, DSC N1 Module, Greifensee, Switzerland) over a temperature range of 25–350 °C at a heating rate of 2 °C/min under a nitrogen gas environment (50 mL/min).
Mechanical properties	TA Plus (Stable Micro Systems, Surrey, UK)	The mechanical properties of the printlets were evaluated using a TX. TA Plus (Stable Micro Systems, Surrey, UK) equipped with a 5-kg load cell with a sensitivity of 0.001 N, operating in compression mode. Samples were compressed using a flat, plane-faced probe with a diameter of 2 mm at the rate of 2 mm/s.
Drug loading content	HPLC (Agilent 1260 Infinity; Agilent Technologies, Waldbronn, Germany)	Drug-loaded printlet was dissolved in 100 mL of deionized water (DI), filtered through a 0.45 μm membrane filter (Zhejiang, China), and diluted with DI water, respectively. The drug concentration was determined by HPLC (Agilent 1260 Infinity; Agilent Technologies, Waldbronn, Germany). The HPLC was performed with 5 μL of injection volume. An isocratic mobile phase composed of 20 mM KH_2PO_4 (pH 2.5) and HPLC-grade methanol (95:5, v/v) was delivered at a flow rate of 1 mL/min for 10 minutes through an Ascentis® C18 column (5 μm, 25 × 4.6 mm; Merck KGaA, Darmstadt, Germany) maintained at 40 °C. Detection of the eluent was carried out at 230 nm. Calibration curve of levodopa with high linear relationship (R^2 = 0.999) was prepared in ranging from 25 to 200 μg/mL in methanol.

Drug releasing properties	HPLC (Agilent 1260 Infinity; Agilent Technologies, Waldbronn, Germany)	Each sample was placed in a 150 mL conical flask containing 100 mL of deionized water and stirred with a magnetic stirrer at 50 rpm at 37 ± 2 °C. Samples were collected at 1, 5, 10, 15, 45, 240, and 1440 min, filtered through 0.45 μm membranes (Zhejiang, China), and diluted with KH_2PO_4, respectively. Drug concentrations were determined by HPLC (Agilent 1260 Infinity) using the same chromatographic conditions as described in Section 2.8 (drug loading). Each test was conducted in triplicate

Table S2. Shaping changing of PPVA, PCom5.0, PCas5.0, PPin2.5, PPin5.0, PPVA, PCom5.0-L, PCas5.0-L, and PPin5.0-L

	Time (min)				
	0	5	10	15	20
PPVA	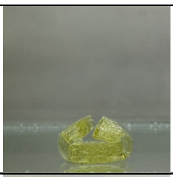	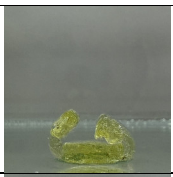	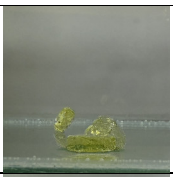	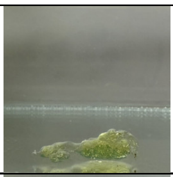	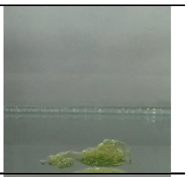
PCom5.0	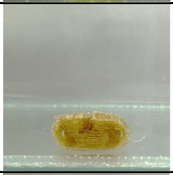	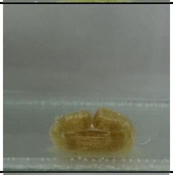	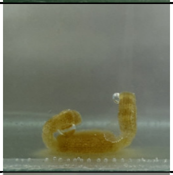	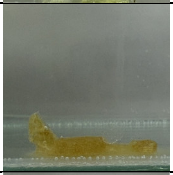	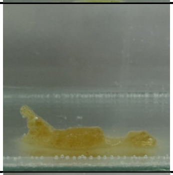
PCas5.0	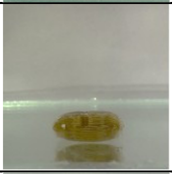	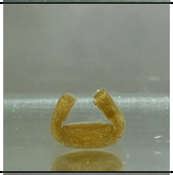	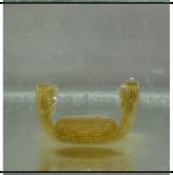	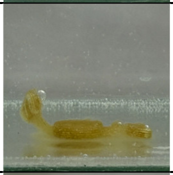	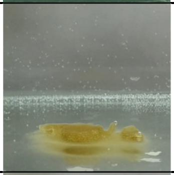
PPin2.5	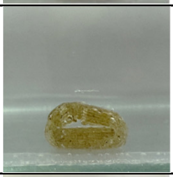	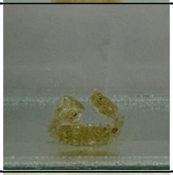	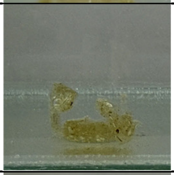	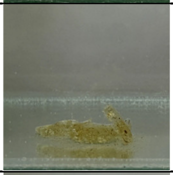	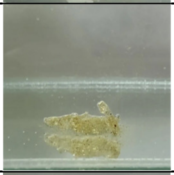
PPin5.0	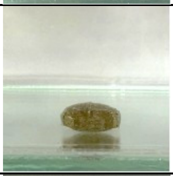	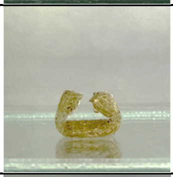	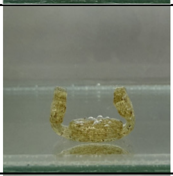	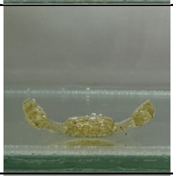	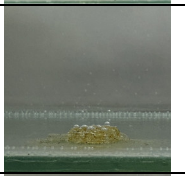
PPVA-L	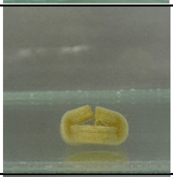	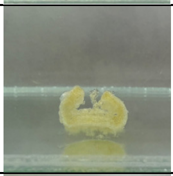	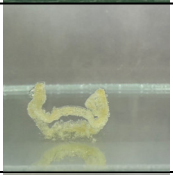	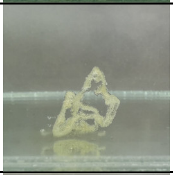	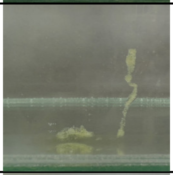
PCom5.0-L	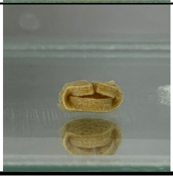	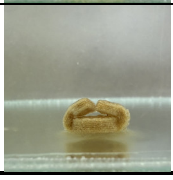	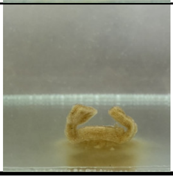	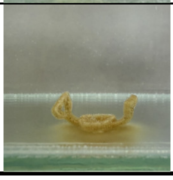	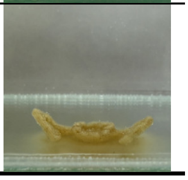

PCas5.0-L	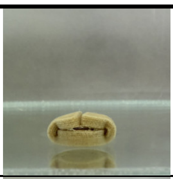	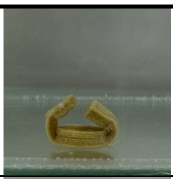	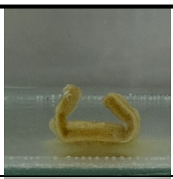	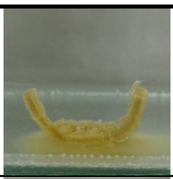	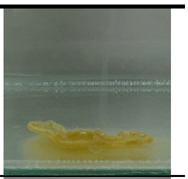
PPin5.0-L	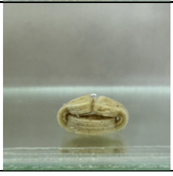	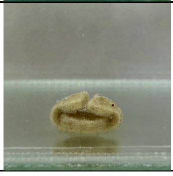	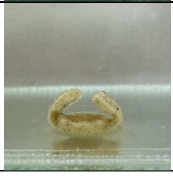	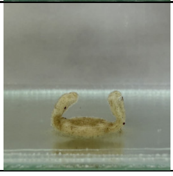	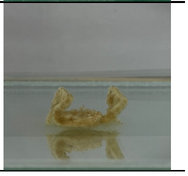
